# Role of Endothelial ADAM17 in Early Vascular Changes Associated with Diabetic Retinopathy

**DOI:** 10.3390/jcm9020400

**Published:** 2020-02-02

**Authors:** Lamiaa Shalaby, Menaka Thounaojam, Amany Tawfik, Junnan Li, Khaled Hussein, Wan Jin Jahng, Mohamed Al-Shabrawey, Hang Fai Kwok, Manuela Bartoli, Diana Gutsaeva

**Affiliations:** 1 Department of Ophthalmology, Medical College of Georgia, Augusta University, Augusta, GA 30912, USA; LSHALABY@augusta.edu (L.S.); mthounaojam@augusta.edu (M.T.); mbartoli@augusta.edu (M.B.); 2 Department of Oral Biology, Dental College of Georgia, Augusta University, Augusta, GA 30912, USA; amtawfik@augusta.edu (A.T.); malshabrawey@augusta.edu (M.A.-S.); hussein.k.nrc@gmail.com (K.H.); 3 Institute of Translational Medicine, Faculty of Health Sciences, University of Macau, Avenida de Universidade, Taipa, Macau SARhfkwok@um.edu.mo (H.F.K.); 4 Department of Petroleum Chemistry, American University of Nigeria, Yola, Nigeria; wan.jahng@aun.edu.ng

**Keywords:** diabetic retinopathy, a disintegrin and metalloproteinase 17, endothelial cells, vascular inflammation, vascular permeability

## Abstract

ADAM17, a disintegrin and metalloproteinase 17, is a transmembrane metalloproteinase that regulates bioavailability of multiple membrane-bound proteins via ectodomain shedding. ADAM17 activity was shown to contribute to a number of vascular pathologies, but its role in the context of diabetic retinopathy (DR) is not determined. We found that expression and enzymatic activity of ADAM17 are upregulated in human diabetic *postmortem* retinas and a mouse model of streptozotocin-induced diabetes. To further investigate the contribution of ADAM17 to vascular alterations associated with DR, we used human retinal endothelial cells (HREC) treated with ADAM17 neutralizing antibodies and exposed to glucidic stress and streptozotocin-induced endothelial ADAM17 knockout mice. Evaluation of vascular permeability, vascular inflammation, and oxidative stress was performed. Loss of ADAM17 in endothelial cells markedly reduced oxidative stress evidenced by decreased levels of superoxide, 3-nitrotyrosine, and 4-hydroxynonenal and decreased leukocyte-endothelium adhesive interactions in vivo and in vitro. Reduced leukostasis was associated with decreased vascular permeability and was accompanied by downregulation of intercellular adhesion molecule-1 expression. Reduction in oxidative stress in HREC was associated with downregulation of NAD(P)H oxidase 4 (Nox4) expression. Our data suggest a role for endothelial ADAM17 in DR pathogenesis and identify ADAM17 as a potential new therapeutic target for DR.

## 1. Introduction

Diabetic retinopathy (DR) is a common complication of diabetes mellitus and the leading cause of preventable blindness in adults [1,2]. The number of people affected by vision impairment due to DR increased between 1995 and 2015 from 1.4 million to 2.6 million, and this number is anticipated to rise to 3.2 million in 2020 [3]. It is recognized that the clinically significant loss of vision in diabetic patients is associated with hyperglycemia-induced vascular abnormalities [4,5,6]. During DR progression, chronic exposure to hyperglycemia can lead to endothelial cell activation, acceleration of reactive oxygen species (ROS) production, increased leukostasis and breakdown of the blood–retinal barrier (BRB) [7,8]. These events lead to progressive alterations of the retinal microvasculature that culminate in formation of degenerate capillaries, micro aneurysms, and contribute to the development of diabetic macular edema and neovascularization in the most advanced disease stages. Chronic subclinical inflammation has been shown to be a key causative factor for the development of microvascular alterations characterizing DR [2].

ADAM17 (a disintegrin and metalloproteinase 17), also referred to as TNFα converting enzyme or TACE, is a type-1 zinc-dependent transmembrane multi-domain metalloproteinase [9,10]. In addition to TNFα, this sheddase is responsible for cleavage and regulation of many structurally and functionally diverse molecules involved in inflammation (reviewed in [11]). These include cytokine receptors (TNFR-I/II, interleukin-6 receptor (IL-6R)), neurotrophins and proapoptotic receptors (p75NTR), chemokines, adhesion molecules such as vascular cell adhesion molecule-1 (VCAM-1) and intercellular adhesion molecule-1 (ICAM-1), and tight junctions proteins such as junction adhesion molecule-A (JAM-A) [9,10,12,13,14,15,16].

Various reports have shown that over-activation of this sheddase is involved in a broad range of pathologic conditions including inflammatory and neurodegenerative diseases [17,18,19,20,21]. Upregulation of ADAM17 has been shown to contribute to diabetic nephropathy [22,23] and, in an animal model of non-obese, insulin-resistant diabetes blocking of ADAM17 activity restored insulin sensitivity [24]. Interestingly, consistent with the hyperactive state of ADAM17, diabetic patients have elevated serum and vitreous levels of soluble factors such as TNFα and TNFα receptors, IL-6 receptor, and adhesion molecules such as ICAM-1 and VCAM-1 [25,26,27]. Most importantly, increased circulating levels of these soluble molecules are strongly associated with progression of DR [25,26,27], thus raising the question of whether and how ADAM17 may contribute to DR pathogenesis especially to diabetes-induced retinal microvascular injury.

Several studies have found a contributing role for ADAM17 in different vascular pathologies [28,29,30,31]. Increased activity of ADAM17 was implicated in vascular remodeling [28], hypoxia-induced impairment of neural vascular barrier [32], abdominal aortic aneurysm [29], and age-related coronary microvascular dysfunction [30]. In retinal endothelial cells, upregulation of ADAM17 activity by high glucose conditions was linked to apoptotic cell death [33]. In addition, ADAM17 has been shown to exert proangiogenic properties [34,35] and contribute to pathological retinal neovascularization [35,36].

Based on the potential contribution of ADAM17 to inflammation and a large number of vascular pathologies, here we sought to investigate the specific contribution of endothelial ADAM17 to DR pathology, a mechanism that has not been previously investigated.

## 2. Experimental Section

### 2.1. Human Samples

*Postmortem* human retinal samples were obtained from the Georgia Eye Bank through their approved research program and used in the present study per protocol approved by the Augusta University Institutional Biosafety Committee. All tissue samples were de-identified prior to receipt; therefore, Institutional Review Board (IRB) approval was not required. According to the available accompanying documentation, DR of unknown severity was present in all diabetic *postmortem* samples. The controls were non-diabetic samples with various co-morbidities disclosed where available. Table S1 summarizes the information about the human samples used in our studies.

### 2.2. Experimental Animals

Care, use, and treatment of all animals were in accordance with the statement of the Association for Research in Vision and Ophthalmology (ARVO) for the humane use of animals in vision science and with protocols approved by Augusta University. Male C57Bl/6J mice were purchased from Jackson Laboratories (Stock No: 000664; Bar Harbor, ME). Endothelial-specific ADAM17 knockout mice were generated by crossing Adam17tm1.2Bbl/J mice (Stock No: 009597; Jackson Laboratories, Bar Harbor, ME, USA), which harbor loxP sites flanking exon2 of ADAM17 with mice expressing Cre recombinase under the control of a Cadh5 promoter (Stock No: 006137; B6.Cg-Tg(Cdh5-cre)7Mlia/J; Jackson Laboratories). After several appropriate crosses, conditional knockout mice with deleted expression of ADAM17 in the vascular endothelial cells (ADAM17Cre-flox mice) and control mice not carrying Cre-transgene (ADAM17flox mice) were generated. Genotype was determined by PCR using tail genomic DNA and KAPA Mouse Genotyping Kit (KAPABiosystem, Wilmington, MA, USA). All strains were tested and proved negative for the presence of retinal degeneration mutations.

### 2.3. STZ Model of Type I Diabetes

Male mice of 8–10 weeks old were made diabetic by intraperitoneal injections of a freshly prepared solution of streptozotocin (STZ; 55 mg/kg of body weight in 100 mM sodium citrate, adjusted to pH 4.5) for 3–5 consecutive days. Diabetes was verified 2 weeks later by measuring blood glucose (hyperglycemia defined as >250 mg/dL). Body weight was measured weekly. Insulin (0−0.2 units of neutral protamine Hagedorn NPH insulin) was given subcutaneously as needed (0–2 times per week) to prevent ketosis without preventing hyperglycemia and glycosuria. Animals were maintained in a hyperglycemic state for 8–10 weeks.

### 2.4. Assessment of Retinal Vascular Permeability

Retinal vascular permeability was assessed by fluorescein angiography using Phoenix Micron III retinal imaging microscope (Phoenix Research Laboratories, Pleasanton, CA, USA). Mice were anesthetized with 2% isoflurane. Pupils were dilated using 1% tropicamide (Bausch & Lomb, Rochester, NY, USA), and Goniovisc 2.5% (hypromellose; Sigma Pharmaceuticals, LLC, Monticello, IA, USA) was applied liberally to retain surface moisture during imaging. Mice were given an intraperitoneal injection of 10% fluorescein sodium (20 μL; Apollo Ophthalmics, Newport Beach, CA, USA). Fluorescent images were taken at constant interval for every mouse studied in each experimental group. In addition, we assessed vascular permeability quantitatively by measuring albumin extravasation to the retinal tissue as described before [37]. Briefly, the mice were deeply anesthetized with ketamine/xylazine (80/12 mg/kg of body weight, respectively). The chest cavity was opened and a 22-gauge perfusion cannula was introduced into the left cardiac ventricle. Drainage was achieved by opening the right atrium. The animals were perfused with phosphate-buffered saline (PBS) to wash out all blood. Retinas then were excised and serum albumin levels were measured in the perfused retinal tissue by Western blot using anti-mouse albumin antibody.

### 2.5. ADAM17 Activity

ADAM17 enzymatic activity in retinal extracts of control and diabetic mice was assessed by using SensoLyte Activity Assay kit (AnaSpec, Fremont, CA, USA) following the manufacturer’s instructions.

### 2.6. Analysis of Leukocyte Adhesion

Leukocyte adhesion to the retinal endothelium was evaluated as described previously [38]. Following the induction of deep anesthesia (ketamine/xylazine, 80 and 12 mg/kg of body weight, respectively), the chest cavity was opened, and a 22-gauge perfusion cannula was introduced into the left ventricle. Drainage was achieved by opening the right atrium. The animals were perfused with 10 mL of warm PBS to wash out non-adherent blood cells. Next, the animals were perfused with 10 mL of fluorescein-labeled concanavalin A (ConA) lectin (40 μg/mL in PBS, pH 7.4; Vector Laboratories, Burlingame, CA, USA) to label the adherent leukocytes and vascular endothelial cells. Residual unbound ConA was removed by perfusion with PBS. The eyeballs were removed and fixed with 4% paraformaldehyde. Retinas were flat-mounted using Fluoromount anti-fading mounting medium (Fisher Scientific, Pittsburg, PA, USA) and examined for epifluorescence using a Zeiss Axioplan-2 microscope (Carl Zeiss, Göttingen, Germany) equipped with the Axiovision program (version 4.7, Carl Zeiss, Göttingen, Germany). The total number of adherent leukocytes in the retinal arterioles, venules, and capillaries was determined.

### 2.7. Retinal Single Cell Suspension Preparation and Flow Cytometry Analysis

Pooled retina samples (3 retinas per group) were incubated in PBS containing DNAse1 and collagenase D (1:1000) at 37 °C for 15 min, centrifuged, and then filtered through 100 μm and 30 μm cell strainers. After washing, the cells were collected by centrifugation and processed for staining with anti-ADAM17-FITC antibody (Bioss, Boston, MA, USA). ADAM17 cell surface expression was analyzed by FACScanto system (BD Biosciences, San Jose, CA, USA). Data were analyzed using FCS Express 4 software (De Novo Software, Los Angeles, CA, USA).

### 2.8. Immunohistochemical Analysis

Immunostaining of retinal sections was performed as described [38]. Mouse eyes were enucleated, embedded in optimal cutting temperature mounting medium (Tissue-Tek, Torrance, CA, USA), frozen on dry ice, and cryostat sectioned (10 µm). Slides were fixed with 4% paraformaldehyde (PFA) and incubated overnight at 4 °C with anti-ADAM17 (1:500; LSBio, Seatle, WA, USA) and anti-cluster of differentiation 31, (CD31; 10 μg/mL; R&D Systems, Minneapolis, MN, USA) antibodies, followed by incubation with appropriate fluorescence-conjugated secondary antibodies (Life Technologies, Eugene, OR, USA). Sections were mounted using Fluoromount anti-fading mounting medium (ThermoFisher Scientific, Waltham, MA, USA) and examined for epifluorescence using a Zeiss Axioplan-2 microscope (Carl Zeiss) equipped with the Axiovision program (version 4.7; Carl Zeiss).

### 2.9. Dihydroethidium (DHE) Staining for Detection of Superoxide

Ten-μm-thick retinal cryosections from control and diabetic mice were brought to room temperature, covered with 2 μM DHE solution and incubated in a light-protected humidified incubator at 37 °C for 20 min. At the end of the incubation, sections were rinsed and mounted using Fluoromount anti-fading mounting medium (ThermoFisher Scientific, Waltham, MA, USA). The images were taken using a Zeiss Axioplan-2 microscope (Carl Zeiss) equipped with the Axiovision program (version 4.7; Carl Zeiss).

### 2.10. Protein Analysis

Proteins were extracted from mouse retinas and human retinal endothelial cells (HREC) as we described previously [39]. The extracted proteins were quantified by using BioRad Protein DC Assay (Bio-Rad, Hercules, CA, USA) and subjected to SDS-PAGE [39]. Then, they were transferred to nitrocellulose membranes, and proteins were blocked and incubated with primary antibodies against ADAM17 (1:1000; Abclonal, Woburn, MA, USA), albumin (1:30,000; Bethyl Laboratories, Montgomery, TX, USA), zonula occluden-1 (ZO-1) (1:1000; ThermoFisher Scientific, Waltham, MA, USA), ICAM-1 (1:500; Abclonal, Abclonal, Woburn, MA, USA), NAD(P)H oxidase 2 (Nox2; 1:800; Abcam, Cambridge, MA, USA), NAD(P)H oxidase 4 (Nox4; 1:500; Abcam, Cambridge, MA, USA), and corresponding horseradish-conjugated secondary antibodies. Actin was used as an internal control. Chemiluminescence-based assay was used for protein detection (SuperSignal West Pico Chemiluminescent Substrate; Pierce Biotechnology, Rockford, IL, USA).

### 2.11. Dot Blot Analysis

Equivalent amount of proteins prepared from mouse retinal and HREC lysates were spotted on nitrocellulose membranes and dried for 5 min at room temperature. The membranes were blocked for 1 h and then probed with either anti-3-nitrotyrosine (3-NT; 1:1000; Cayman, Ann Arbor, Michigan,) or anti-4-hydroxynonenal (4-HNE; 1:1000; Abcam, Cambridge, MA, USA) antibodies overnight at 4 °C. After washing, the membranes were incubated with corresponding horseradish peroxidase-conjugated secondary antibody. The immuno-positive spots were visualized using chemiluminescence-based assay (SuperSignal West Pico Chemiluminescent Substrate; Pierce Biotechnology, Rockford, IL, USA).

### 2.12. Cell Culture and Treatments

HREC were purchased from Cell Systems Corporation (Kirkland, WA, USA) and cultured in a growth medium at 37 °C in a humidified atmosphere of 5% CO_2_ in air as suggested by the manufacturer. HREC were used between passages 3–7. Prior to all experiments, HREC monolayers were serum starved for 6–8 h. Cells were exposed to 5 mM D-Glucose (NG, normal glucose) and 25 mM D-glucose (HG, high glucose) for 24–72 h in the presence of 0.5 µM anti-ADAM17 blocking antibody D1(A12) or normal human IgG (R&D Systems) [40]. Cells treated with 25 mM L-glucose (LG) were used as the osmotic control. Treated cells were used for evaluation of leukocyte adhesion and measurements of trans-endothelial resistance (TER) or harvested for analysis by quantitative polymerase chain reaction (qPCR) and Western blotting analysis.

### 2.13. Quantitative PCR

Total RNA was isolated from HREC using TRI Reagent (Sigma-Aldrich, St. Louis, MO, USA) according to the manufacturer’s protocol. cDNA was prepared using iScript™cDNA Synthesis Kit (Bio-Rad, Hercules, CA, USA). Amplification of ADAM17 Forward 5′-CCCCGATGTGAGCAGTTT-3′, Reverse 5′-AATCAAGCTTCTCGAGTCTCTG-3′ mRNA was performed using Power SYBR green PCR master mix (Applied Biosystems, Foster City, CA, USA). The relative mRNA abundance was determined by normalizing to hypoxanthine phosphoribosyltransferase 1 (HPRT-1) using the ΔΔCt method. The conditions used for the PCR were as follows; 95 °C for 3 min (1 cycle) and 94 °C for 20 s, 55 °C for 30 s, and 72 °C for 40 s (40 cycles). The thermal cycler StepOne™ Real-Time PCR System (Applied Biosystems; Foster City, CA, USA) was used for qPCR, and the data were analyzed using iCycler Thermal Cycler software (Applied Biosystems, Foster City, CA, USA).

### 2.14. Leukocyte Adhesion Assay

Peripheral blood mononuclear cells (PBMC) preparation: mouse PBMC were isolated from sodium heparin collected mouse blood by 1077/1119 Histopaque double-gradient density centrifugation (Histopaque; SigmaAldrich, St. Louis, MO, USA). The viability of cells was determined by Trypan blue exclusion assay. Cells were counted and the concentration was adjusted to 2 × 10^7^ cells/mL. Fifty microliters of the cell suspension (1 × 10^6^ cells) was added to 12 × 75-mm tube and stained with phycoerythrin (PE) rat anti-mouse CD45 antibody (BD Biosciences)-conjugated monoclonal antibody for 40 min at 4 °C. Cells were washed and collected for the adhesion assay. Cell adhesion assay: HREC were grown in Falcon^®^ 4-well culture slides and treated with HG in the presence of 0.5 µM ADAM17 blocking antibody D1(A12) or normal human IgG (isotype control) for 48 h. After end of treatment, cells were rinsed with PBS and incubated with PBMC (5 × 10^5^ cells per chamber). After a 30-min incubation, the non-adherent cells were gently washed with PBS and leukocytes adherent to HREC monolayer were fixed with 4% PFA. Sections were mounted using Fluoromount anti-fading mounting medium with DAPI (ThermoFisher Scientific) and examined for epifluorescence using a Zeiss Axioplan-2 microscope (Carl Zeiss). CD45-positive cells were counted microscopically in ten randomly selected fields for each group. The number of adherent cells was normalized to the number of HREC (number of leukocytes per 100 HREC).

### 2.15. Measurements of TER of EC Monolayer

TER in HREC monolayers treated with NG and HG in the presence of ADAM17 blocking antibody D1(A12) or normal human IgG (isotype control) was measured using an electrical cell substrate impedance sensing system (ECIS; Applied Biophysics, Troy, NY, USA). HREC were seeded on 8W10E arrays and TER was monitored for up to 72 h. Resistance values for multiple wells were normalized to the identical starting resistance value at time zero and data from three wells were averaged and presented as normalized resistance versus time.

### 2.16. Statistical Analysis

Values are mean ± standard error (SE). The data were analyzed by Student’s *t*-test or Mann–Whitney rank sum test using a computer-based software package GraphPad Prism 7.0 (San Diego, CA, USA). *p*-values less than 0.05 were considered significant. For all data, “*n*” represents the number of animals per group or the number of independent cell experiments. Total number of mice used in the study was: C57Bl/6J *n* = 35, ADAM17Cre-flox *n* = 47, ADAM17flox *n* = 43.

## 3. Results

### 3.1. ADAM17 Expression and Activity Are Increased in Human and Mouse Diabetic Retinas

**Figure 1:**
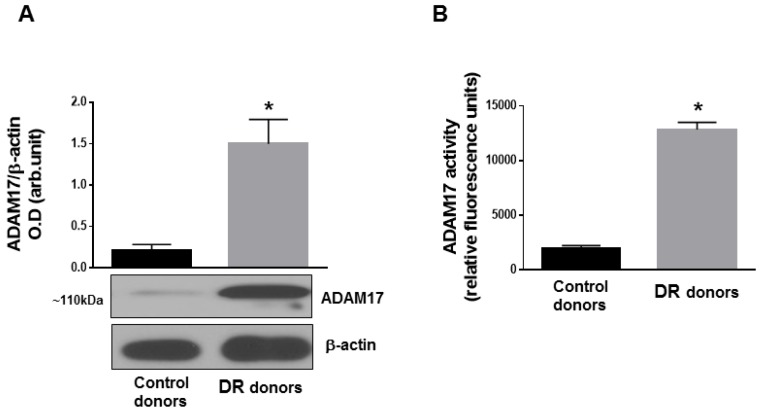
A disintegrin and metalloproteinase 17 (ADAM17) expression and activity in human postmortem retinal tissue. (**A**) Representative blots and densitometry analysis of ADAM17 expression and (**B**) ADAM17 enzymatic activity measured by fluorimetric assay in postmortem human retinas from normoglycemic (control) donors and donors with diabetic retinopathy (DR). Data presented as mean ± SE. * *p* < 0.01. *n* = 6 in each group.

To understand the contribution of ADAM17 to DR pathology, we first measured expression and activity of ADAM17 in human postmortem control and diabetic retinas. As shown in Figure 1, protein expression of ADAM17 determined by Western blot analysis (Figure 1A) and activity of the enzyme evaluated by fluorimetric assay (Figure 1B) were significantly elevated in postmortem diabetic retinas compared to control donor tissues. Next, we determined whether ADAM17 expression and activity were upregulated in retinas of STZ-mice.

**Figure 2:**
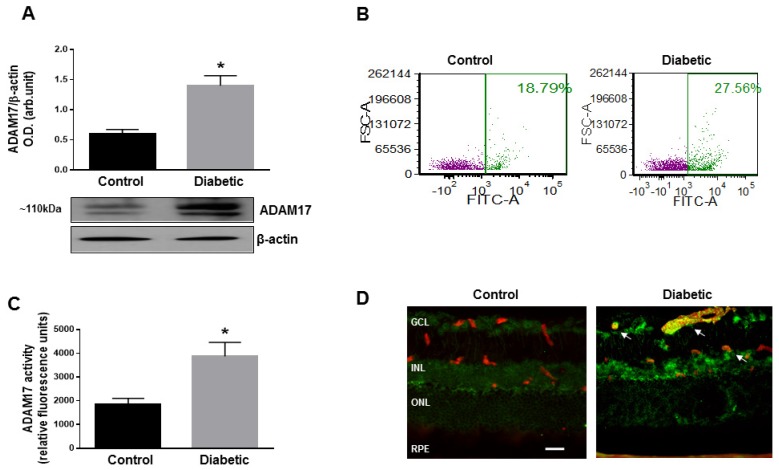
ADAM17 expression in the mouse retina. (**A**) Representative blots and densitometry analysis of ADAM17 expression in normoglycemic (control) and diabetic C57Bl/6J mice (diabetic) mice at 8 weeks of diabetes. Data presented as mean ± standard error (SE). * *p* < 0.01. *n* = 5 per group. (**B**) Flow cytometric analysis of cell surface expression of ADAM17 in retinal single cell suspension from control and diabetic C57Bl/6J mice at 8 weeks of diabetes. *n* = 3 pooled retinas per group. (**C**) ADAM17 enzyme activity measured by fluorimetric assay in control and diabetic retinas of C57Bl/6J mice. Data presented as mean ± SE. * *p* < 0.01. *n* = 5 per group. (**D**) Representative images of ADAM17 specific immunoreactivity (green) in retinas of control and diabetic C57Bl/6J mice at 8 weeks of diabetes. Anti-CD31 antibody (red) was used to co-label endothelial cells. Arrows indicate coexpression of ADAM17 and CD31. GCL, ganglion cell layer; INL, inner nuclear layer; ONL, outer nuclear layer; RPE, retinal pigment epithelial cells. Scale bar, 50 µm.

Similar to human postmortem tissues, in mouse retinas, hyperglycemia induced a significant increase in the expression levels of ADAM17 (*p* < 0.01 compared to control age-matched normoglycemic mice; Figure 2A). As the processes of ADAM17 maturation and activation involve its translocation to the cell plasma membrane [41], we further measured the cell surface expression of ADAM17 in retinal slurry of control normoglycemic and diabetic mice by flow cytometry. As shown in Figure 2B, the hyperglycemic milieu promoted translocation of ADAM17 to the plasma membrane. Increased translocation of ADAM17 correlated with increased activity of ADAM17 in retinas of diabetic mice measured by fluorimetric assay (Figure 2C). Immunoreactivity to ADAM17 assessed by imunofluorescence staining of retinal cryosections was localized to the ganglion and inner nuclear cell layers (GCL and INL, respectively) and was significantly elevated in the diabetic retina (Figure 2D). Notably, significant upregulation of ADAM17 was observed in the vasculature of diabetic animals as evidenced by positive co-staining of retinal cryosections with the endothelial cell marker CD31 (Figure 2D; arrows).

### 3.2. Endothelial ADAM17 Contributes to Hyperglycemia-Induced Vascular Permeability

**Figure 3:**
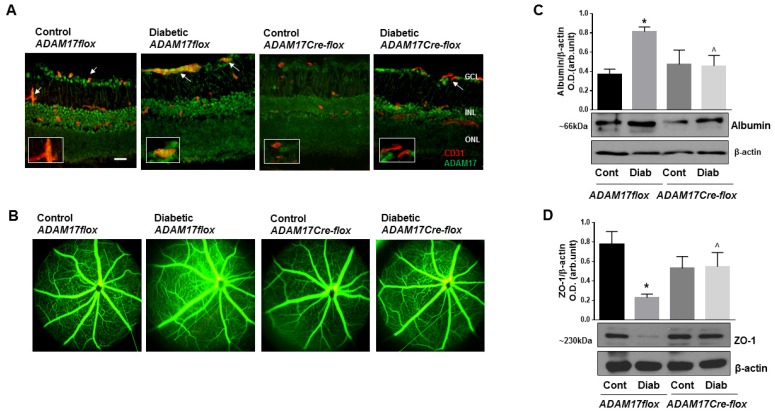
Diabetes-induced retinal vascular leakage is reduced in mice with conditional inactivation of ADAM17 in vascular endothelium. (**A**) Representative images of ADAM17-specific immunoreactivity (green, ADAM17; red, CD31; yellow, merging, white arrows) in retinal cryosections of normoglycemic and diabetic ADAM17Cre-flox and ADAM17flox mice at 10 weeks of diabetes. Inserts are magnified images of representative blood vessels. Scale bar, 50 µm. (**B**) Fluorescein angiography from control normoglycemic and diabetic ADAM17flox and ADAM17Cre-flox mice. *n* = 5 in each group. (**C**) Western blot analysis assessing albumin protein levels in retinal extracts of perfused mice from control normoglycemic and diabetic ADAM17flox and ADAM17Cre-flox mice. Data presented as mean ± SE. * *p* < 0.01 diabetic vs. control. ^ *p* < 0.05 diabetic ADAM17flox vs. diabetic ADAM17Cre-flox mice. *n* = 5 in each group. (**D**) Representative blots and densitometry analysis of ZO-1 expression from control normoglycemic and diabetic ADAM17flox and ADAM17Cre-flox mice. Data presented as mean ± SE. * *p* < 0.01 diabetic vs. control. ^ *p* < 0.05 diabetic ADAM17flox vs. diabetic ADAM17Cre-flox mice. Cont, control; Diab, diabetic. *n* = 5 per group.

Upregulation of ADAM17 in retinal vasculatures prompted us to further investigate the contribution of vascular ADAM17 to the pathogenesis of DR. For this purpose, we generated endothelial-specific ADAM17 knockout mice by crossing mice carrying floxed alleles of ADAM17 with a strain of Cre mice in which Cre-recombinase is expressed under the control of endothelial VE-Cadherin promoter [42,43]. Breeding of ADAM17flox with ADAM17Cre-flox mice gave rise to offspring with the expected Mendelian ratio (~55% ADAM17flox and ~45% ADAM17Cre-flox mice) and, overall, we did not observe differences in postnatal mortality in all the experimental groups. ADAM17Cre-flox and control ADAM17flox mice were rendered diabetic by five consecutive injections of STZ. Blood glucose concentrations and body weights of diabetic ADAM17flox and ADAM17Cre-flox mice were similar (Table S2). Immunofluorescence staining of retinal cryosections demonstrated no expression of ADAM17 in endothelial cells of mice with conditional inactivation of the enzyme (ADAM17Cre-flox) compared to their control littermates (ADAM17flox; Figure 3A). Diabetes increased the expression of ADAM17 in endothelial cells of ADAM17flox mice but not in ADAM17Cre-flox mice (Figure 3A; arrows) further confirming the efficiency of ADAM17 knockdown in endothelial cells in these mice.

Blood–retinal barrier breakdown accompanied by vascular leakage is a key feature of DR. To determine the contribution of endothelial ADAM17 in this pathologic process, we assessed vascular barrier function in control and endothelial ADAM17 knockout mice at 10 weeks of diabetes qualitatively using fluorescein angiography and quantitatively by measuring albumin extravasation in retinal tissue by Western blot (Figure 3B,C). Our results showed no difference in vascular permeability between control normoglycemic ADAM17flox and ADAM17Cre-flox mice (Figure 3B). As expected, diabetes-induced vascular leakage in ADAM17flox mice was evidenced by the appearance of areas of hyperfluorescence (Figure 3B). This effect was significantly attenuated in ADAM17Cre-flox diabetic mice (Figure 3B). Parallel measurements of albumin extravasation in retinas of normoglycemic and diabetic mice revealed a significant reduction in the amount of extravascular albumin in diabetic ADAM17Cre-flox mice compared to diabetic control ADAM17flox mice (Figure 3C). Changes in retinal vascular permeability may result from alterations of the endothelial cell tight junction (TJ) complex. We evaluated whether inactivation of ADAM17 in vascular endothelium affected the expression of TJ protein ZO-1, which is a critical determinant of barrier formation [44]. As expected, diabetes reduced protein levels of ZO-1 in diabetic ADAM17flox mice compared to control normoglycemic mice (*p* < 0.01); however, the knockdown of ADAM17 helped preserve the levels of ZO-1 in diabetic ADAM17Cre-flox mice (*p* < 0.05 diabetic ADAM17flox vs. diabetic ADAM17Cre-flox mice; Figure 3D). These data suggest that endothelial ADAM17 activity may contribute to diabetes-induced BRB dysfunction by affecting expression of TJ proteins.

### 3.3. Lack of Endothelial ADAM17 Diminishes Hyperglycemia-Induced Retinal Inflammation

**Figure 4:**
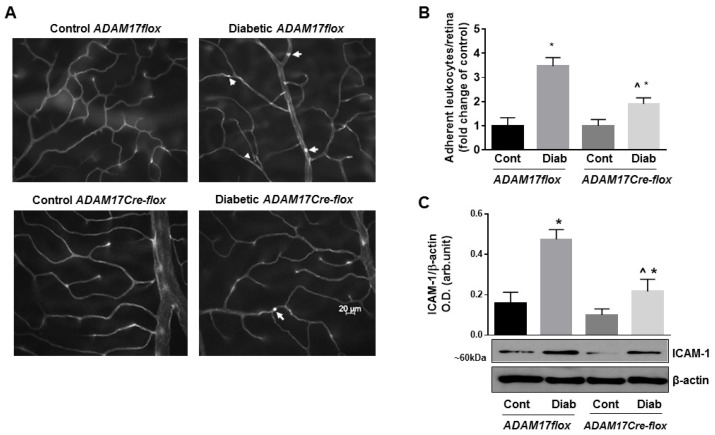
Diabetes-induced retinal leukostasis is reduced in mice with conditional inactivation of ADAM17 in vascular endothelium. (**A**) Representative images of flat-mounted retinas from control normoglycemic and diabetic ADAM17flox and ADAM17Cre-flox mice stained with ConA to identify leukocytes adherent to retinal microvessels (white arrows). Scale bar: 20 μm. (**B**) Quantification of adherent leukocytes. Data are expressed as adherent leukocytes per retina and presented as a fold change from corresponding controls. * *p* < 0.05 diabetic vs. corresponding control. ^ *p* < 0.05 diabetic ADAM17flox vs. diabetic ADAM17Cre-flox mice. *n* = 5 in each group. (**C**) Representative blots and densitometry analysis of ICAM-1 protein levels in retinal extracts from control normoglycemic and diabetic ADAM17flox and ADAM17Cre-flox mice. Data presented as mean ± SE. * *p* < 0.05 diabetic vs. control. ^ *p* < 0.01 diabetic ADAM17flox vs. diabetic ADAM17Cre-flox mice. Cont, control; Diab, diabetic. *n* = 5 per group.

Low-grade chronic inflammation contributes to DR pathogenesis and could contribute to BRB breakdown. To identify, whether ADAM17 activity contributes to vascular inflammation in DR, we analyzed leukocyte adhesion in retinal vasculature of diabetic ADAM17flox mice and mice lacking the expression of ADAM17 in endothelial cells following in situ labeling with ConA [8]. Lack of endothelial ADAM17 did not affect the number of adherent leukocytes in non-diabetic mice (Figure 4A,B). Retinas of diabetic ADAM17flox mice showed more than 3-fold increase in the number of adherent leukocytes compared with age-matched non-diabetic controls (*p* < 0.01; Figure 4A,B). Knockdown of ADAM17 significantly reduced the number of adherent leukocytes in retinas of diabetic mice (*p* < 0.05 diabetic ADAM17flox vs. diabetic ADAM17Cre-flox mice; Figure 4A,B). As expected, diabetes-induced expression of the leukocyte adhesion molecule ICAM-1 in retinas of ADAM17flox mice (*p* < 0.001 vs. corresponding control; Figure 4C), but knockdown of vascular ADAM17 significantly reduced retinal ICAM-1 protein levels in diabetic ADAM17Cre-flox mice (*p* < 0.01 diabetic ADAM17flox vs. diabetic ADAM17Cre-flox mice; Figure 4C). These data are further confirming reduced inflammatory responses in diabetic mice lacking endothelial ADAM17.

### 3.4. Endothelial ADAM17 Knockdown Diminishes Hyperglycemia-Induced Oxidative Stress

**Figure 5:**
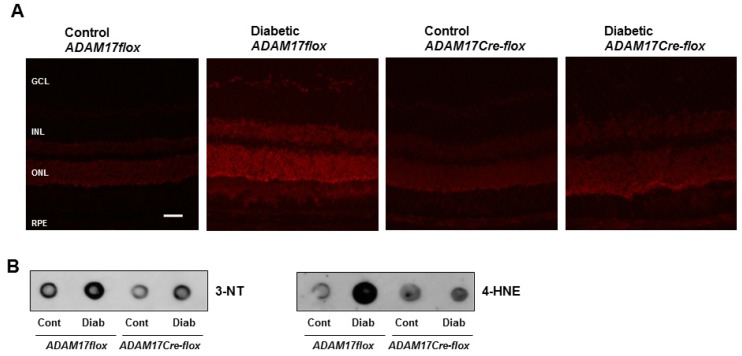
Diabetes-induced oxidative stress is decreased in mice with conditional inactivation of endothelial ADAM17. (**A**) Representative images of DHE-stained retinal sections from control normoglycemic and diabetic ADAM17flox and ADAM17Cre-flox mice. *n* = 3. Scale bar: 50 µm. (**B**) Dot blot analysis of 4-HNE and 3-NT in retinal tissue from control normoglycemic and diabetic ADAM17flox and ADAM17Cre-flox mice. Cont, control; Diab, diabetic. *n* = 3.

Hyperglycemia-induced oxidative stress is an important contributing factor to retinal inflammation [45]. To test whether upregulated ADAM17 contributes to diabetes-induced oxidative stress in the retina, we measured superoxide production by dihydroethidium (DHE) staining. Probing retinal sections from non-diabetic ADAM17flox and ADAM17Cre-flox mice with DHE showed minimal staining (Figure 5A). Diabetes promoted a significant amplification of DHE fluorescence reactivity in diabetic ADAM17flox mice, but this effect was significantly abrogated in mice lacking endothelial expression of ADAM17 (Figure 5A). Next, we measured the formation of 3-nitrotyrosine (3-NT), a putative marker of peroxinitrite, and 4-hydroxynonenal (4-HNE), a highly reactive lipid peroxidation product, in diabetic ADAM17flox and ADAM17Cre-flox mice by dot blot analysis. As demonstrated in Figure 5B, the diabetic milieu promoted the generation of both 3-NT and 4-HNE in retinas of ADAM17flox mice. Inactivation of endothelial ADAM17 markedly decreased these effects of hyperglycemia (Figure 5B) suggesting that ADAM17 activity contributes to hyperglycemia-induced oxidative stress.

### 3.5. Glucidic Stress Upregulates Expression of ADAM17 in HREC

**Figure 6:**
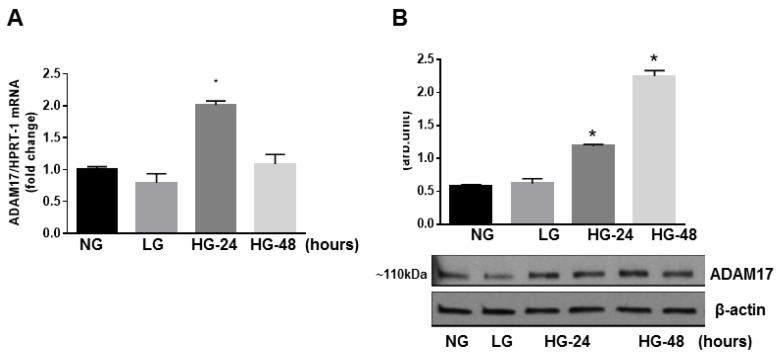
Effect of high glucose on ADAM17 expression in vitro. (**A**) QPCR analysis of ADAM17 mRNA. (**B**) Representative blots and densitometry analysis of ADAM17 protein expression in HREC treated with 5 mM D-glucose (NG, normal glucose) or 25 mM D-glucose (HG, high glucose) for 24–48 h. L-glucose (LG, 25 mM) served as the osmotic control). * *p* < 0.001 NG vs. HG. Data presented as mean ± SE. *n* = 3.

To further dissect, at the molecular level, the effects of endothelial ADAM17 knockdown seen in diabetic mice, we studied the effects of ADAM17 blocking on isolated retinal endothelial cells of human origin (HREC) subjected to glucidic stress. First, we confirmed that ADAM17 is upregulated in high glucose conditions. We measured mRNA and protein expression of ADAM17 in HREC exposed to normal glucose (NG; 5 mM), high glucose (HG; 25 mM), or L-glucose (LG; 25 mM), which served as the osmotic control. We found that high glucose exposure (24 h) promoted a significant upregulation of ADAM17 mRNA as measured by qPCR (Figure 6A). Transcriptional upregulation of ADAM17 was associated with an increase in ADAM17 protein levels that was also sustained 48 h post high glucose treatment (Figure 6B). These data are in agreement with findings of others who showed upregulation of ADAM17 enzymatic activity in retinal endothelial cells in high glucose conditions [33].

### 3.6. ADAM17 Contributes to High Glucose-Induced Retinal Endothelial Cell Permeability

**Figure 7:**
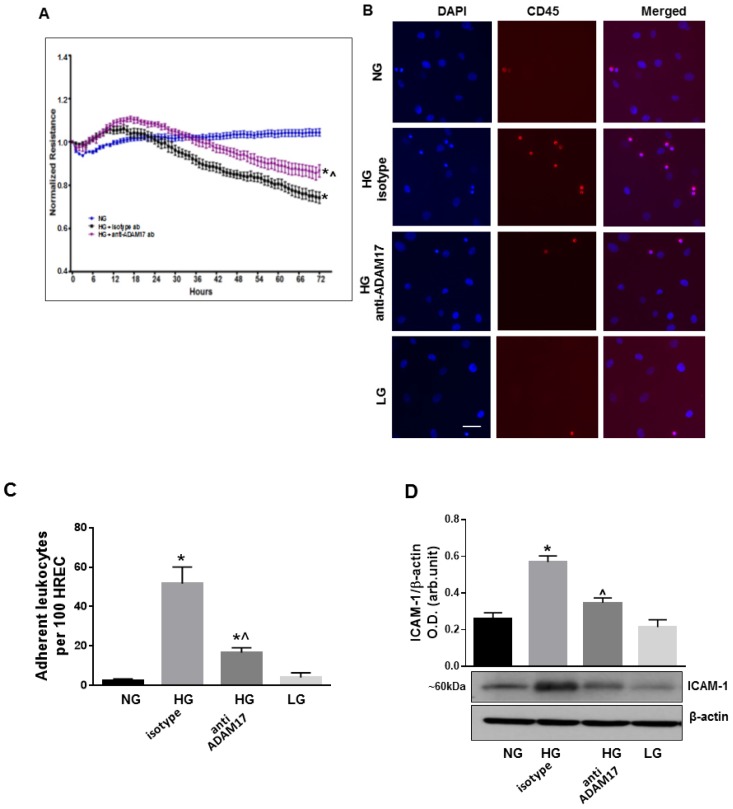
Effects of ADAM17 blocking antibody on high glucose-induced endothelial cell permeability, leukocyte adhesion and ICAM-1 expression. (**A**) TER in HREC monolayers treated with HG in the presence of ADAM17 blocking antibody or control isotype antibody using ECIS. Data presented as mean ± SE. * *p* < 0.05 NG vs. HG. ^ *p* < 0.05 HG + isotype vs. HG + anti-ADAM17 antibody. *n* = 3. (**B**) Representative images of leukocytes adherent to HREC exposed to HG in the presence of 0.5 µM ADAM17 blocking antibody D1(A12) or control isotype antibody. Scale bar, 50µm. (**C**) Quantitative analysis of leukocyte adhesion. Data are expressed as a number of adherent cells per 100 HREC. * *p* < 0.05 NG vs. HG. ^ *p* < 0.05 HG + isotype vs. HG + anti-ADAM17 antibody. *n* = 3. (**D**) Representative blots and densitometric analysis of ICAM-1 expression in HREC treated with HG in the presence of ADAM17 blocking antibody or control isotype antibody. Data presented as mean ± SE. * *p* < 0.05 NG vs. HG + isotype antibody. ^ *p* < 0.05 HG + isotype vs. HG + anti-ADAM17 antibody. *n* = 4.

Next, we analyzed the effects of ADAM17 blocking on retinal paracellular permeability in HREC using ECIS methodology. HREC were treated with NG or HG (0–72 h) in presence of ADAM17 blocking antibody D1(A12) or control isotype antibody. The assessment of the basal TER in the treated groups showed no significant difference (data not shown). Treatment with HG lowered the TER in HREC monolayers over the course of high glucose treatment as compared to control normoglycemic cells (Figure 7A). Blocking of ADAM17 activity partially halted the effects of glucidic stress as demonstrated by reduced loss of TER in treated cells (*p* < 0.01 HG+anti-ADAM17 ab vs. HG+isotype ab; Figure 7A).

### 3.7. Blocking of ADAM17 Diminishes High Glucose-Induced Leukocyte Adhesion *in Vitro*

We also examined interactions of leukocytes with HREC exposed to high glucose and treated with ADAM17 blocking antibody D1(A12) or control isotype antibody. We found that glucidic stress (HG; 48 h) promoted a significant increase in the number of leukocytes adherent to HREC treated with control antibody (*p* < 0.001 vs. NG; Figure 7B,C). Blocking of ADAM17 activity substantially decreased the number of adherent cells (*p* < 0.001 vs. HG; Figure 7B,C). Decreased HG-induced adhesion of leukocytes to HREC in the presence of ADAM17 blocking antibody was associated with downregulation of ICAM-1, a key mediator of leukocyte adhesion to the endothelium (Figure 7D).

### 3.8. Blocking of ADAM17 Reduces Oxidative Stress in High Glucose-Stimulated HREC

**Figure 8:**
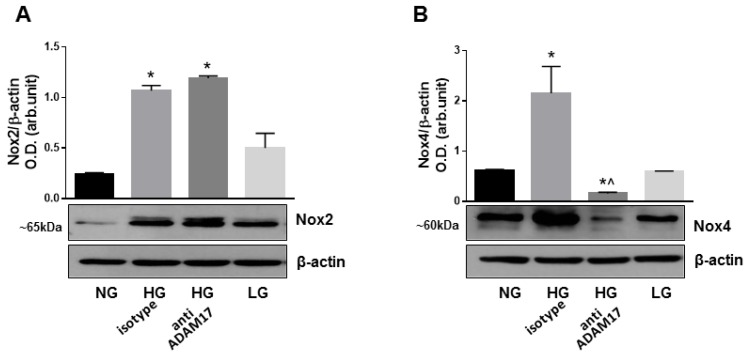
Effects of ADAM17 blocking on expression of Nox2 and Nox4 in high glucose-stimulated HREC. Representative blots and densitometry analysis of Nox2 and Nox4 expression in HREC treated with HG in the presence of ADAM17 blocking antibody or control isotype antibody. Data presented as mean ± SE. * *p* < 0.05 NG vs. HG. ^ *p* < 0.05 HG + isotype vs. HG + anti-ADAM17 antibody. *n* = 4.

Hyperglycemia-induced oxidative stress has been shown to play a key role in DR pathogenesis and in the proinflammatory responses associated with diabetes-induced retinal microvascular dysfunction [45,46]. Nox2 and Nox4, members of NADPH oxidase family of proteins, play an essential role in vascular endothelial dysfunction in diabetes [45,46]. Recent studies have also linked increased activation of ADAM17 to Nox4 induction [22]; thus, suggesting that this mechanism could be operational in diabetes-induced retinal microvascular injury. Therefore, we examined expression of Nox2 and Nox4 in HREC exposed to high glucose in the presence of ADAM17 blocking antibody. Western blotting analysis showed that high glucose upregulated the expression of both Nox2 and Nox4 in HREC treated with isotype control antibody (Figure 8A,B). Blocking of ADAM17 by specific neutralizing antibody did not affect the expression of Nox2, but significantly reduced the expression of Nox4 in high glucose-stimulated cells (Figure 8A,B). These data suggest that ADAM17 activity contributes to Nox4 activation in retinal endothelial cells in response to glucidic stress.

## 4. Discussion

DR is characterized by multiple pathological processes, such as chronic low-grade inflammation, increased vascular permeability, accelerated retinal cell death, and neovascularization [2,47,48,49]. In this study, we sought to determine the contribution of ADAM17 to early vascular alterations associated with DR pathogenesis. Using mice with conditionally inactivated ADAM17 and cultured human retinal microvascular endothelial cells, we were able to dissect the effects of endothelium-derived ADAM17 in diabetes-induced vascular alterations in early experimental diabetes. We demonstrated that hyperglycemia-induced upregulation of endothelial ADAM17 contributed to numerous key responses linked to DR pathogenesis, including oxidative stress, inflammation, and vascular barrier breakdown. Most importantly, the direct relevance of this finding to human DR is supported by the presence of overactive ADAM17 in human diabetic postmortem retinal tissue.

A number of signaling events implicated in the pathogenesis of DR results from altered balance between soluble and surface-expressed molecules originating from different retinal cells [50,51,52]. Many of these factors exist as trans-membrane molecules that become released from the cell surface via ectodomain shedding mediated by members of the ADAM family of proteins, in particular, ADAM17 [9,16]. For example, previous studies have implicated ADAM17 in the regulation of several substrates involved in the pathogenesis of DR including TNFα, TNFRI/II, IL-6R, p75NTR, and ICAM-1 [9,10,12,13,14,15,16]. Our data are consistent with previous reports showing that ADAM17 contributes to multiple vascular pathologies [28,29,30]. In particular, studies in brain microvascular endothelial cells demonstrated an important contribution of ADAM17 to vascular barrier impairment in response to hypoxia [32]. In agreement with these studies, we found that genetic ablation of endothelial ADAM17 is sufficient to ameliorate vascular barrier function in retinas of diabetic mice. The increase in retinal vascular permeability is associated with changes in the expression of tight junction proteins and distribution of these proteins at the cell borders. Previous findings suggest a potential role of ADAM17 in regulation of endothelial tight and adherence junction organization [32,53]. In our experimental conditions, decreased vascular leakage in retinas of diabetic ADAM17 knockout mice was associated with the recovery of the expression of ZO-1 expression, a key regulator of cellular tight junctions [44].

Vascular inflammation and leukostasis are early events in DR development that can lead to disorganization of endothelial tight junctions and the BRB breakdown [54]. Consistent with the ability of ADAM17 to regulate inflammatory responses, we also found a marked reduction in leukocyte adhesion in diabetic mice lacking endothelial ADAM17 as well as in high glucose-stimulated HREC treated with ADAM17 neutralizing antibodies. Interestingly, these effects were associated with the reduction in levels of the leukocyte adhesion molecule ICAM-1. This molecule is a known substrate of ADAM17 in physiological conditions [55]; however, our data suggest that, in the diabetic milieu, this sheddase could directly control ICAM-1 expression through alternate mechanisms that should be further studied. One of such mechanism could possibly involve ROS-mediated activation of NF-κB [56,57]. Increased generation of ROS by two isoforms of NADPH oxidase, Nox2 and Nox4, has been linked to endothelial dysfunction in the diabetic retina [45,46]. The observed reduction of oxidative stress in mice with inactive endothelium-derived ADAM17 indicates that hyperglycemia-induced ADAM17 activity contributes to increased ROS generation in the diabetic retina. To investigate potential mechanisms explaining this effect, we assessed the expression of Nox enzymes in retinal endothelial cells treated with high glucose in the presence or absence of ADAM17-specific neutralizing antibodies. The results of these studies showed that, although high glucose increased the expression of both Nox2 and Nox4, inhibition of ADAM17 markedly reduced Nox4 levels. The latter is the major isoform of NADPH oxidase in retinal microvascular endothelial cells [46]. It was reported previously, that Nox4 exists in a conformation that allows for spontaneous transfer of electrons from NADPH to FAD and confers the enzyme a constitutive activity; therefore, Nox4-regulated ROS production is governed by changes in its expression levels [58]. Overall, our findings imply that upregulation of Nox4 expression and subsequent ROS production in the hyperglycemic milieu is mediated at least in part by an ADAM17-dependent mechanism and directly implicates this sheddase as a regulator of endothelial cells redox responses. Interestingly, the role of ADAM17 as an upstream regulator of Nox4 was recently reported to be involved in extracellular matrix accumulation during diabetic nephropathy [22]. The mechanism by which ADAM17 regulates Nox4 oxidase expression in hyperglycemia is still under investigation and remains to be determined.

In summary, the conditional inactivation of ADAM17 in endothelial cells provides the first evidence for a critical role of ADAM17 in retinal vascular alterations associated with diabetes. Our results also suggest that interventions directed at the regulation of ADAM17 activity can be beneficial in preventing and/or alleviating vascular complications associated with DR.

## Supplementary Materials

The following are available online at https://www.mdpi.com/2077-0383/9/2/400/s1, Table S1: Postmortem Donor History, Table S2: Average body weight and blood glucose levels in control and diabetic mice.
