# Supplementary material for: Role of Endothelial ADAM17 in Early Vascular Changes Associated with Diabetic Retinopathy
Source: J Clin Med. 2020 Feb 2;9(2):400. doi: 10.3390/jcm9020400 (PMC7073770; doi:10.3390/jcm9020400)
Supplement: Supplementary file 1 [file jcm-09-00400-s001.pdf]

Table S1. *Postmortem* Donor History

Donors	Experimental Group	Age	Sex	Significant Medical Conditions	Ocular History
1	Control/non-diabetic	51	M	HTN, non-invasive eye surgery	
2	Control/non-diabetic	52	M	HTN, high cholesterol	
3	Control/non-diabetic	46	F	Obesity	
4	Control/non-diabetic	52	M		
5	Control/non-diabetic	56	F		
6	Control/non-diabetic	41	F		
7	Control/non-diabetic	37	M		
8	Control/non-diabetic	56	M	HTN, lupus	
9	Control/non-diabetic	48	F	Obesity, CAD	
10	Control/non-diabetic	68	M	Coronary artery bypass graft, HTN	
11	Diabetic	62	F	Dialysis, HTN, stroke	DR
12	Diabetic	60	F	Obesity	DR, laser OS
13	Diabetic	67	M	IDDM 10yrs, toe amputation, dialysis	DR, laser OS
14	Diabetic	71	M	IDDM, dialysis, Parkinson's Disease, altered mental state	DR, glaucoma
15	Diabetic	73	M	IDDM 25yrs, dialysis, toe amputation	DR, laser OS & OD
16	Diabetic	57	F	ESRD, IDDM 10yrs, obesity	DR
17	Diabetic	49	M	Leg amputation	DR
18	Diabetic	52	M	Dialysis	DR
19	Diabetic	72	M	NDDM 10yrs, pancreatic cancer	Edema
20	Diabetic	68	F	Alzheimer's, right lung mass	DR

HTN, hypertension; IDDM, insulin-dependent diabetes mellitus; NDDM, noninsulin-dependent diabetes mellitus; ESRD, end-stage renal disease; OS, oculus sinister (left eye); OD, oculus dexter (right eye)

Table S2. Average body weight and blood glucose levels in control and diabetic mice

	ADAM17flox		*ADAM17Cre-flox*	
	Control	Diabetic	Control	Diabetic
Body Weight	35.5±0.82	28.8±0.82*	35.0±0.71	26.8±0.28*
Blood glucose	114.3±13.89	496.2±31.59**	116.0±8.97	451.2±30.82**

*p<0.001 compared to corresponding control; **p<0.0001 compared to corresponding control.
